# Templating metallocycles with a macrocycle: synthesis, structures and magnetic studies of {Cr_11_M_2_} complexes†

**DOI:** 10.1039/d2dt03368b

**Published:** 2022-11-30

**Authors:** Rajeh Alotaibi, Adam Brookfield, Jonathan M. Fowler, George F. S. Whitehead, Selena J. Lockyer, Grigore A. Timco, David Collison, Jürgen Schnack, Richard E. P. Winpenny

**Affiliations:** Department of Chemistry, The University of Manchester Oxford Road Manchester M13 9PL UK richard.winpenny@manchester.ac.uk; Department of Chemistry, King Saud University Riyadh 11451 Saudi Arabia; Faculty of Physics, P.O. Box 100131, Bielefeld University D-33501 Bielefeld Germany

## Abstract

Addition of 1,4,8,11-tetrazacyclotetradecane (cyclam) to a reaction that produces octametallic rings when simpler amines are used, produces {Cr_11_M_2_} “pretzels” (M = Zn^II^ or Cu^II^) where the cyclam coordinates to the M^II^ ion which then sits at the centre of a twelve-metal macrocycle. Magnetic studies were fitted using the finite-temperature Lanczos method (FTLM), and the results demonstrate that exchange interactions are transferable from previous exchange-coupled Cr^III^ rings.

## Abstract 2

Two new metal rings are reported, grown around a [M(cyclen)]^2+^ macrocyclic complex (M = Zn^II^ or Cu^II^, cyclen = 1,4,7,10-tetrazacyclododecane).

## Graphical abstract

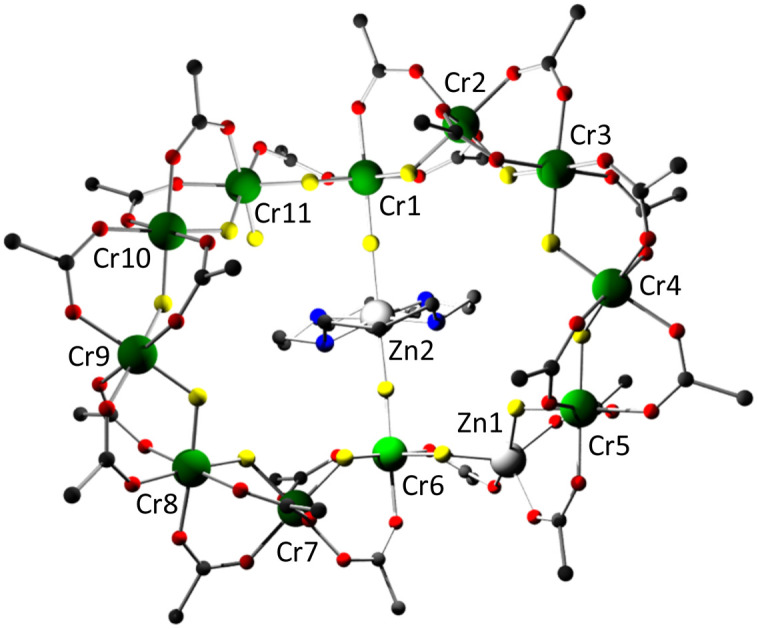

## Introduction

Template synthesis was one of the earliest methods used to synthesise macrocyclic ligands.^1^ Later, Raymond and others^2–6^ have shown that addition of templating counter-ions can be used to control the structure of polymetallic complexes, for example converting metal double-helices into metal tetrahedra. We have previously reported the templating of heterometallic rings, using dialkylammonium cations such as di-*n*-propylammonium to form {Cr_7_M^II^} rings^6,7^ (M^II^ = a divalent 3d-metal ion such as Ni^II^) where the more sterically demanding cation di-i-propylammonium leads to {Cr_8_M^II^} rings.^8,9^ More unusually, use of 1,4,7-triazacyclonanone (tacn) leads to coordination of the macrocycle to the divalent metal and the resulting [M^II^(tacn)_2_]^2+^ dication templates the formation of a {Cr_8_M^II^_2_} metallocycle.^10^

Using 1,4,7,10-tetrazacyclododecane (cyclen) also leads to formation of a coordination complex with the M^II^ ion, but as the cyclen only occupies four of the six sites of the metal coordination sphere, the metal becomes part of an open chain structure, either [{Ni(cyclen)}_2_Cr_12_NiF_20_(O_2_C^*t*^Bu)_22_]^10^ or [Cu(H_2_O)(cyclen)]_2_[Cr_24_Cu_5_{Cu(cyclen)}_2_F_40_(O_2_C^*t*^Bu)_50_],^11^ depending on whether Ni^II^ or Cu^II^ is used. The other common tetradentate macrocycle is 1,4,8,11-tetrazacyclotetradecane (cyclam), and we have now examined what happens with this larger macrocyclic ligand.

The reactions we report involve Zn^II^ and Cu^II^ as the divalent metal ion. The basic metal carbonate was dissolved in pivalic acid at 140 °C in the presence of a small amount of water. Cyclam was then added to the solution and heated for a further 30 minutes. The solution was then allowed to cool to room temperature and hydrated chromium fluoride was added to the solution and then the reaction was heated to 160 °C for five hours. After cooling to room temperature, the product was precipitated by adding acetone. Column chromatography was needed to purify the crude product, with [Cr(O_2_C^*t*^Bu)_2_]_8_ a major by-product. Crystals could be grown from Et_2_O/MeCN (3 : 1) by slow evaporation.

**Fig. 1:**
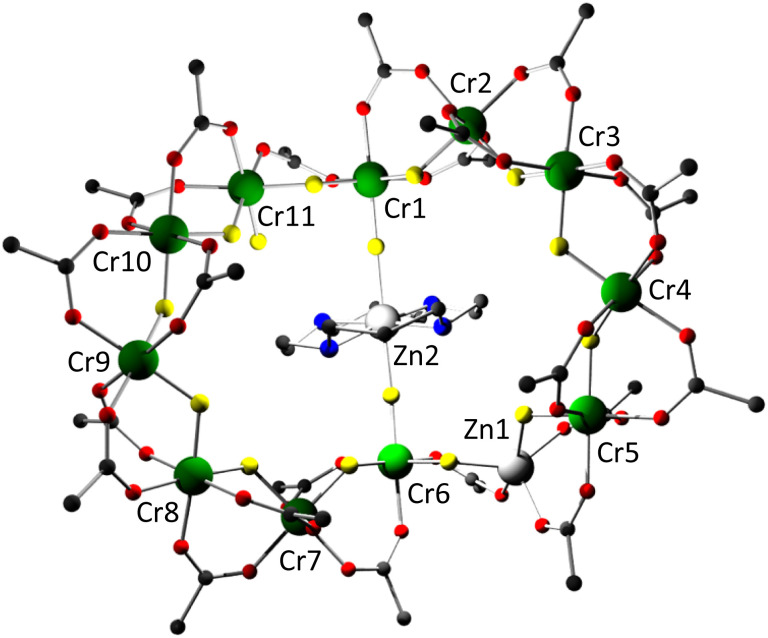
The structure of 1 in the crystal. Colour scheme: Cr, green; Zn, white; F, yellow; N, blue; O, red, C, black. Methyl groups on pivalates and H-atoms excluded for clarity.

[M(cyclam)][Cr_11_MF_15_(O_2_C^*t*^Bu)_22_] (M = Zn^II^1 or Cu^II^2) crystallise with the [M(cyclam)]^2+^ cation at the centre of a twelve-metal metallocycle (Fig. 1). There are two molecules in the asymmetric unit in 2, with the [Cu(cyclam)]^2+^ fragment disordered in 1. The metal site within the cyclam has two fluorides completing its coordination sphere. The second divalent metal ion is found within the twelve-metal metallocycle and is predominantly five-coordinate. The other eleven metal sites are six-coordinate, and it is the coordination number that leads us to assign the sites as Cr^III^ with no disorder between the metals in these eleven sites.

The chromium sites have three distinct coordination spheres. Two (Cr1 and Cr6) are bound to three fluorides and three O-donors from pivalate with *fac*-geometry. Each of these fluoride bridges to another metal. One chromium (Cr11) is also bound to a *fac*-3F, 3O coordination sphere, but one of the fluorides here is terminal. The remaining Cr sites have 2F, 4O coordination as is normally found in this family of heterometallic rings.^7^ The central divalent metal is six-coordinate (4N, 2F) with a *trans* octahedral geometry, while the five-coordinate site is bound to 2F and 3O donors in 1 and 2. In 1 the site has a *τ* value = 0.39,^12^ indicating almost mid-way between square pyramidal and trigonal bipyramidal, while in 2, where the five-coordinate Cu^II^ site requires two disordered sites in the model the *τ* values are 0.13 and 0.22, indicating a geometry closer to square pyramidal in both models.^12^

The structures are not planar; there is a marked fold at the Cr1–M2–Cr6 edge. In 1 the mean plane between the {Cr_5_} chain and this edge is compared with the mean plane of the {Cr_4_Zn} chain and this edge, then the fold is 60°. In 2 the fold is 31° in one molecule and 34° in the second.

There is limited H-bonding in the structures. There is a single H-bond from the terminal fluoride to a cyclam N–H; this is either 2.61 or 2.69 Å in the two molecules of 2 and longer in 1 at 2.97 Å. The N–H of the cyclams have contacts of around 3 Å to the apical F-atoms bound to the metal site. In 1 one of these distances falls to 2.67 Å.

In both structures there is significant disorder around the five-coordinate site within the ring. In 1 there is a partial occupancy atom in a sixth-coordination site and the anisotropic displacement parameter (ADP) is too large if the site is exclusively zinc; we therefore included a part-weight chromium in the site bound to a terminal fluoride. In 2 in one of the two molecules in the asymmetric unit a terminal fluoride also has a large ADP. This may be associated with disorder in the position of the [Cu(cyclam)]^2+^ dication. More details are given in the ESI.†

**Fig. 2:**
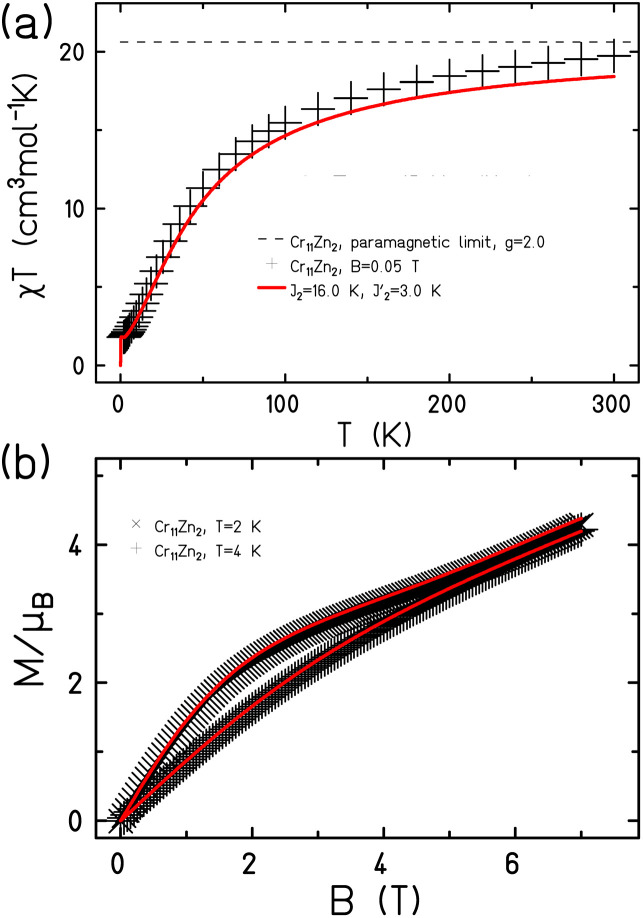
The magnetic behaviour of 1 shown (a) as *χT*(*T*) and (b) *M*(*B*). Measured data as black symbols, fits as red curves.

**Fig. 3:**
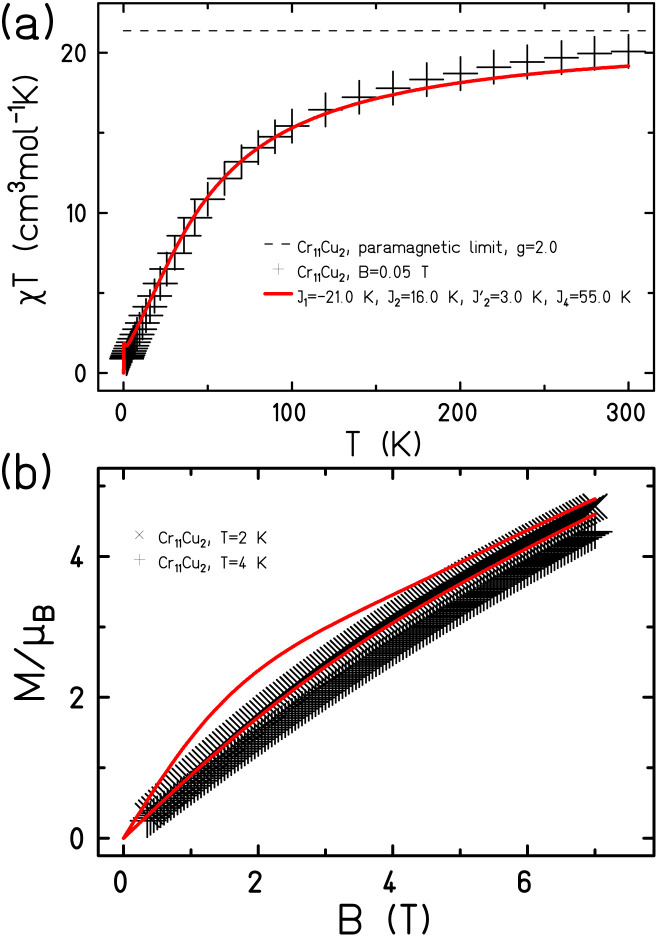
The magnetic behaviour of 2 shown (a) as *χT*(*T*) and (b) *M*(*B*). Measured data as black symbols, fits as red curves.

The magnetic properties of 1 and 2 show predominantly anti-ferromagnetic exchange between the paramagnetic metal centres (Fig. 2 and 3). As the compounds are complex with a large number of paramagnetic centres, we made several assumptions about the likely size of the exchange interactions.

**Fig. 4:**
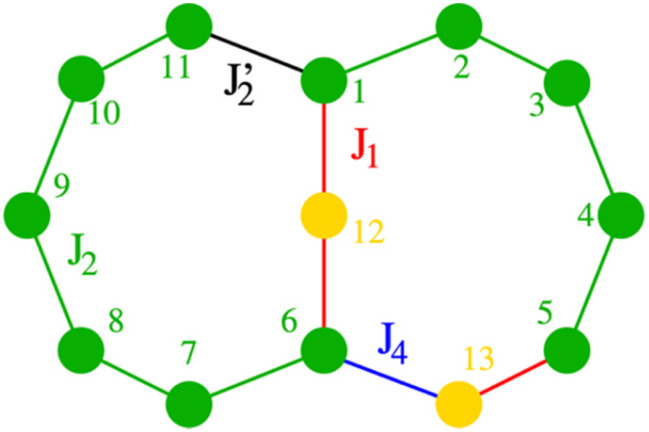
Numbering of spin centres (Cr, green; M, yellow) and exchange interactions. In 1*J*_1_ and *J*_4_ are not present.

For 1 there are eleven Cr^III^ ions in a chain with only two distinct types of bridging. For ten of the eleven edges the Cr⋯Cr interaction is bridged by a single fluoride and two pivalates; we have measured this multiple times previously^7^ and we assign this as *J*_2_ (Fig. 4, to make it directly comparable to values given in ref. 11). We set this as 16 K in Hamiltonian (1); in this Hamiltonian this is an anti-ferromagnetic exchange. The Cr1⋯Cr11 edge is bridged by a single fluoride and a single pivalate and we have not seen this previously. We assign this as *J*′_2_ in Hamiltonian (1).1
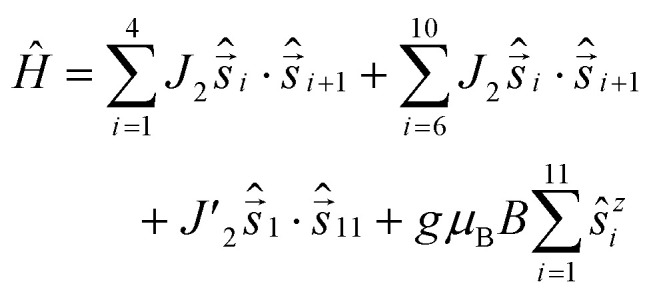


Setting *g* = 2.0 we find *J*′_2_ = 3.0 K, with a good fit of both variable temperature susceptibility (Fig. 2a) and low temperature magnetisation (Fig. 2b).

Given the similarity of the structure, the magnetic model for 2 only needs to add in two further parameters: *J*_1_ which is the exchange between the central Cu^II^ and Cr1 or Cr6; this is through a single fluoride bridge that lies on the *z*-axis of the Cu^II^ ion. This is close to orthogonal to the t_2g_ orbitals of the Cr^III^ ions with which the copper interacts. The second further parameter is *J*_4_ which we use to model the Cr6⋯Cu2 edge. We decided to model the Cr5⋯Cu2 edge with *J*_1_ to maintain minimal variables.

This gives Hamiltonian (2):2
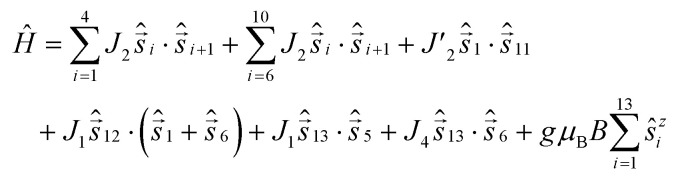
where spins 12 and 13 denote Cu1 and Cu2 respectively.

A very good fit of the measured data is achieved with *J*_1_ = −21.0 K and *J*_4_ = 55.0 K (Fig. 4). All fits have been performed using the finite-temperature Lanczos method (FTLM).^13,14^

The assumptions made here are substantial, *e.g.*, *g* = 2.0 for 1 is possibly a little high, while for 2 two *g*-values could be used for the Cr^III^ and Cu^II^ sites. The major assumption however is that the main exchange present, between two Cr sites bridged by a fluoride and two carboxylates, is an invariable interaction.

**Fig. 5:**
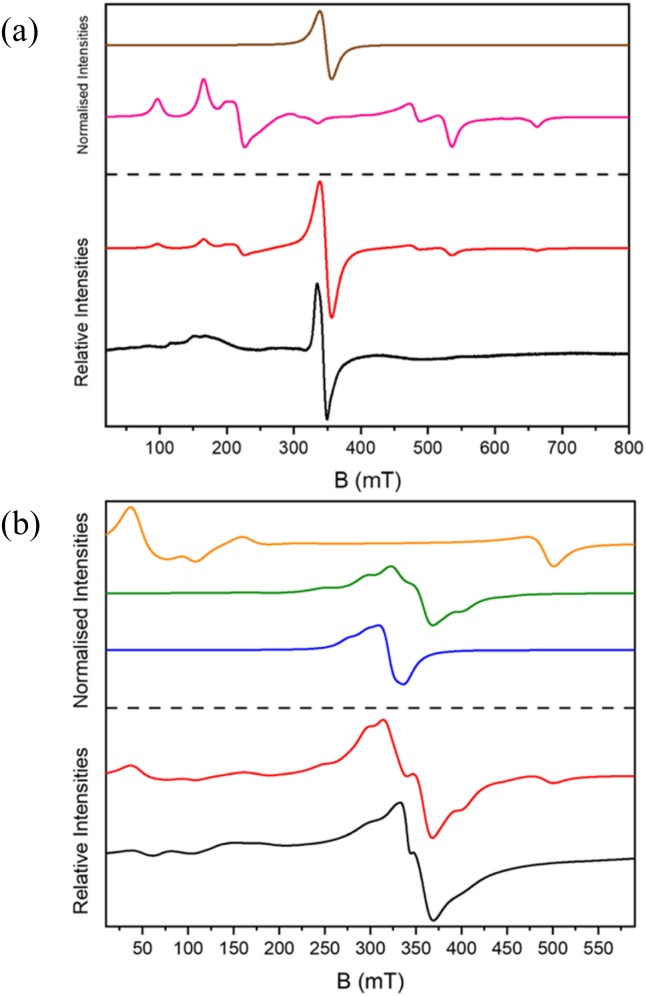
The X-band EPR spectra of 1 and 2. In (a) the spectrum measured at 9.388583 GHz (black), *S* = 5/2 state (brown), *S* = 3/2 state (pink) and simulation weighted at 2 : 1 for the 3/2 : 5/2 states (red). In (b) the spectrum measured at 9.388738 GHz (black), *S* = 1 state (orange), *S* = 2 state (green), *S* = 1/2 (blue), and simulation with equal weights for these states in red.

The X- and Q-band EPR spectra for 1 and 2 are broad and contain some confirmatory information (Fig. 5 and Fig. S1–S3†). For both there is an intense feature at around 350 mT at X-band, with many weaker features spreading from 0–800 mT.

The magnetic model for 1 gives an *S* = 3/2 ground state, with an *S* = 5/2 excited state. We have modelled the EPR spectra as the sum of an *S* = 3/2 state with *D* = −0.34, *E* = −0.02 cm^−1^ and an *S* = 5/2 state with *D* = −0.003 cm^−1^ (Fig. 5a). For 2 we have modelled the X-band spectrum as an isolated *S* = 1/2 due to Cu^II^, and for spin states, *S* = 2 with *D* = −0.003 cm^−1^ and *S* = 1 with *D* = 0.35 cm^−1^ (Fig. 5b). At Q-band features appear that we model with an additional *S* = 1 state (*D* = 1.27 cm^−1^). Given the size of the molecule, there is a danger in over-interpreting the data.

There are two noteworthy results from this work. The first is the extraordinary structural and magnetic consistency in these compounds based on the {–CrF(O_2_C^*t*^Bu)_2_} repeating unit. The magnetic data for 1 and 2, and previously for [Cu(H_2_O)(cyclen)]_2_[Cr_24_Cu_5_{Cu(cyclen)}_2_F_40_(O_2_C^*t*^Bu)_50_] can be fitted with no variation in the predominant *J*_CrCr_ exchange. This transferability is related to the structural rigidity: analysing the structures compared with many other structures containing {Cr_6_} chains^15^ we find all the metric parameters are consistent (Table S3†), *e.g.* the Cr–F–Cr angle 123.2° in 1 and 2 and in {Cr_6_} chains 124.1° and in both cases all the Cr–F–Cr angles lie in the range 122 to 125°.

Secondly, varying from cyclen, which leaves two *cis*-sites vacant on an octahedral metal to cyclam, which leaves two *trans*-sites vacant has an influence that locally is predictable, but which has a dramatic effect on the final structure. The *trans*-coordination to the [M(cyclam)]^2+^ leads to a pinching in of the metallocycle, where the *cis* coordination to [M(cyclen)]^2+^ allows formation of open chains.^8,11^

## Author contributions

RA performed the synthetic chemistry, with advice from GAT. AB and RA measured the magnetometry, advised by DC. SJL measured and simulated the EPR data. GFSW solved and refined the X-ray structures. JS modelled the magnetic data. DC and REPW devised the project. The manuscript was written by DC, JS and REPW with input from all authors.

## Conflicts of interest

There are no conflicts to declare.

## Supplementary Material

DT-052-D2DT03368B-s001

DT-052-D2DT03368B-s002
