# Supplementary material for: Templating metallocycles with a macrocycle: synthesis, structures and magnetic studies of {Cr11M2} complexes
Source: Dalton Trans. 2022 Nov 30;52(1):20–3. doi: 10.1039/d2dt03368b (PMC9764323; doi:10.1039/d2dt03368b)
Supplement: DT-052-D2DT03368B-s001 [file DT-052-D2DT03368B-s001.pdf]

Electronic Supplementary Material (ESI) for Dalton Transactions.
This journal is © The Royal Society of Chemistry 2022

Supporting information

Templating metallocycles with a macrocycle: synthesis, structures and magnetic studies of $\{Cr_{11}M_2\}$ complexes

Rajeh Alotaibi,[a] Adam Brookfield,[a] Jonathan M. Fowler,[a] George F. S. Whitehead,[a] Selena J. Lockyer,[a] Grigore A. Timco,[a] David Collison,[a*] Jürgen Schnack[b] and Richard E. P. Winpenny[a*]

Contents:

A. **Synthesis** 2

B. **Crystallography** 3

C. **Magnetic studies and modelling** 6

D. **EPR spectroscopy** 7

E. **References** 10

Synthesis

All reagents were obtained from Aldrich. The syntheses of complexes were carried out in Erlenmeyer Teflon® FEP flasks supplied by Fisher.

[Zn(cyclam)][$Cr_{11}ZnF_{15}(O_2C^tBu)_{22}$] 1: $2ZnCO_3.3Zn(OH)_2$ (1.13 g, 2.10 mmol), deionized water (1 g, 55 mmol), and $^tBuCO_2H$ (15 g, 147 mmol) were heated to 140 ºC with stirring for 1 h. Cyclam (0.8 g, 4.0 mmol) was added to the mixture and it was stirred for a further 30 mins. The reaction was cooled to room temperature and $CrF_3.4H_2O$ (5.0 g, 28 mmol) was added to the solution and the reaction was stirred at 160 ºC for 5 h. The solution was cooled to room temperature, acetone (100 mL) was added, and the reaction stirred overnight. The product was filtered and washed with a large quantity of acetone. The product was extracted into THF (200 mL), filtered and the filtrate's solvent was removed *in vacuo*, affording the title product as a green solid. The product was purified by column chromatography on silica gel using toluene as an eluent to remove the impurity {Cr_8} until a clear solution was obtained. Then, the polarity was increased by mixing toluene and ethyl acetate (10:1 v/v) to remove the second band, which is the desired product. This ratio of mixed solvent was added until the second band was collected and evaporated to dry under reduced pressure. Yield 0.20 g (2.3%, calculated from the available Cr). Elemental analysis (%): calculated for $C_{120}H_{222}Cr_{11}F_{15}N_4O_{44}Zn_2$: Cr 16.76, Zn 3.83, C 42.23, H 6.56, N 1.64; found: Cr 15.50, Zn 3.58, C 41.87, H 6.98, N 1.48. The product was crystallized from a Et_2O/MeCN (3:1 v/v) mixture by slow evaporation of the solvents and gave X-ray quality crystals in two days.

[Cu(cyclam)][$Cr_{11}CuF_{15}(O_2C^tBu)_{22}$] 2: Compound **2** was synthesised by a similar procedure as compound **1**, but by using basic copper carbonate (1.13 g, 5.1 mmol) instead of $2ZnCO_3.3Zn(OH)_2$. Yield 0.52 g (6.1%, calculated from the available Cr). Elemental analysis (%):

calculated for $C_{120}H_{222}Cr_{11}Cu_2F_{15}N_4O_{44}$: Cr 16.34, Cu 3.63, C 43.57, H 6.62, N 1.60; found: Cr 15.10, Cu 3.43, C 43.61, H 6.64, N 1.47. The product was crystallized from a Et_2O/MeCN (3:1 v/v) mixture by slow evaporation of the solvents and gave X-ray quality crystals in two days.

B. Crystallography

Data for **1** were collected on an Agilent Supernova 4-circle diffractometer with a microfocus MoKα (λ=0.71073Å) source and an Eos CCD detector at a temperature of 150 K. Data for **2** were collected using a dual source Rigaku FR-X rotating anode diffractometer with VariMAX™ optics and a HyPix-6000HE detector, using CuK_α (λ=1.54184Å) at a temperatures of 150 K. All data were and reduced using CrysAlisPro v41.[S1] Absorption correction was performed using empirical methods (SCALE3 ABSPACK) based upon symmetry-equivalent reflections combined with measurements at different azimuthal angles.[S1] The structures were solved using ShelXT and refined against all F^2 values using ShelXL implemented through Olex2 v1.5.[S2,S3]

Both compounds show significant disorder. In **1** the whole structure had to be split into two parts from the need to split some of the metal sites e.g. Cr8/Cr8A. For five-coordinate Zn site within the ring and the ADP for this site is large. There is additional electron density in the position for a sixth ligand. The site was therefore modelled as a mixed Zn-Cr site, which results in much more amenable thermal parameters. We included the sixth coordination site as a fluoride and we tidy the site occupancy of the fluoride to the Cr occupancy (as 5 coordinate Cr(III) is rare).

This results in Zn being present when Cr is not in PART 2, resulting in two different Zn coordination environments. The Zn was therefore split 50:50 into parts 1 and 2 to complete the connectivity table, while Cr was modelled in part 3 with the bonds to the nearest neighbours

added separately. Overall, this is not entirely satisfactory, but it is the only model we could create that would make chemical sense; The Cr needs an F to charge balance. This would fit better as an O but that would mean it was either a neutral water (unlikely given the proximity of the hydrogen bonds), a terminal OH (highly unusual) or an oxide (but the bond distance is too long. The bond distance is also too long for F, but this could be attributed to poor precision in the region being modelled.).

In addition, F6 and F6A was refined to have similar distances, which has resulted in a more satisfactory refinement of their thermal parameters.

For **2** there are two molecules in the asymmetric unit. In one of the molecules F8 has large ADPs and refines well as a 100% occupied oxygen. However, if it were an oxygen it would be either water, OH or O. Water would result in unbalanced charges and unsatisfactory hydrogen bonding to the cation. Terminal OH is unlikely and the bond distance is too long for O. If it were partially occupied F, given that the site refines very well as 100% Cr, the charges would not balance. Therefore, we settled as a 100% fluoride with a large ADP.

Crystallographic data and refinement parameters are given in Table S1. CCDC 2211253-2211254 contains the supplementary crystallographic data for this paper. These data can be obtained free of charge *via* www.ccdc.cam.ac.uk/conts/retrieving.html (or from the Cambridge Crystallographic Data Centre, 12 Union Road, Cambridge CB21EZ, UK; fax: (+44)1223-336-033; or deposit@ccdc.cam.ac.uk).

Table S1: Crystal data for compounds **1** and **2**

	**1**	**2**
Empirical formula	$C_{126.8}H_{240.1}Cr_{11.27}F_{15.27}N_4O_{46.26}Zn_{1.73}$	$C_{121}H_{223.5}Cr_{11}Cu_2F_{15}N_{4.5}O_{44}$
Formula weight	3550.23	3429.61
Temperature/K	150.06(14)	150.00(10)
Crystal system	triclinic	triclinic
Space group	P-1	P-1
a/Å	20.2660(6)	21.8291(5)
b/Å	21.0542(6)	29.6847(7)
c/Å	24.7966(6)	31.7603(6)
α/°	82.820(2)	71.668(2)
β/°	77.658(2)	73.433(2)
γ/°	62.886(3)	70.962(2)
Volume/Å^3	9195.3(5)	18081.3(8)
Z	2	4
ρ_{calc}g/cm^3	1.282	1.260
μ/mm^{-1}	6.194	0.942
F(000)	3717.0	7160.0
Radiation (Å)	CuKα (λ = 1.54184)	Mo Kα (λ = 0.71073)
2Θ range for data collection/°	3.65 to 152.162	5.172 to 42.982
Index ranges	$-25 \le h \le 24, -26 \le k \le 26, -28 \le l \le 31$	$-22 \le h \le 22, -30 \le k \le 26, -32 \le l \le 27$
Reflections collected	119512	78802
Independent reflections	36868 [R_{int} = 0.0539, R_{sigma} = 0.0558]	41387 [R_{int} = 0.0393, R_{sigma} = 0.0813]
Data/restraints /parameters	36868/21713/3581	41387/20734/3942
Goodness-of-fit on F^2	1.014	1.029
Final R indexes [I>=2σ (I)]	R_1 = 0.0678, wR_2 = 0.1948	R_1 = 0.0672, wR_2 = 0.1643
Final R indexes [all data]	R_1 = 0.0921, wR_2 = 0.2153	R_1 = 0.1080, wR_2 = 0.1921
Largest diff. peak/hole / e Å^{-3}	1.14/-0.61	1.46/-0.81

C. Magnetic measurements

Variable-temperature (2.0–300 K) direct current (dc) magnetic susceptibility measurements under an applied field of 1000 G and variable-field (0–7.0 T) magnetization measurements at low temperatures (2.0 and 4 K) were carried out for **1** and **2** constrained in eicosane with a Quantum Design MPMS-XL7 SQUID magnetometer. The susceptibility data were corrected for the diamagnetism of the constituent atoms, the eicosane and the sample holder.

The finite-temperature Lanczos method (FTLM)[S4,S5] was used to calculate the magnetic properties of both compounds. FTLM combines a Krylov space representation of the partition function with a Monte-Carlo average of the trace and achieves quasi exact results up to Hilbert space dimensions of about 10^{11}. An average *g*-value of 2.00 was assumed for all calculations. Up to 250 Lanczos steps were executed per random vector in the largest Hilbert subspaces. For each simulation 100 random vectors were used, and the S^z-symmetry was applied. The best fits of data are shown in the main manuscript.

D. EPR spectroscopy

Continuous wave Q-band (~34 GHz) and X-band (~9.5 GHz) EPR spectra were recorded with a Bruker EMX580 spectrometer. The continuous wave data were collected on polycrystalline powders at 5 K using liquid helium cooling. All continuous wave spectra were field corrected using a ‘Strong Pitch’ standard (g = 2.0028) and all powder samples were checked for any polycrystalline nature, by measuring multiple random rotations of the sample tube. Spectral simulations were performed using the EasySpin 5.2.30 simulation software[S6] unless otherwise stated.

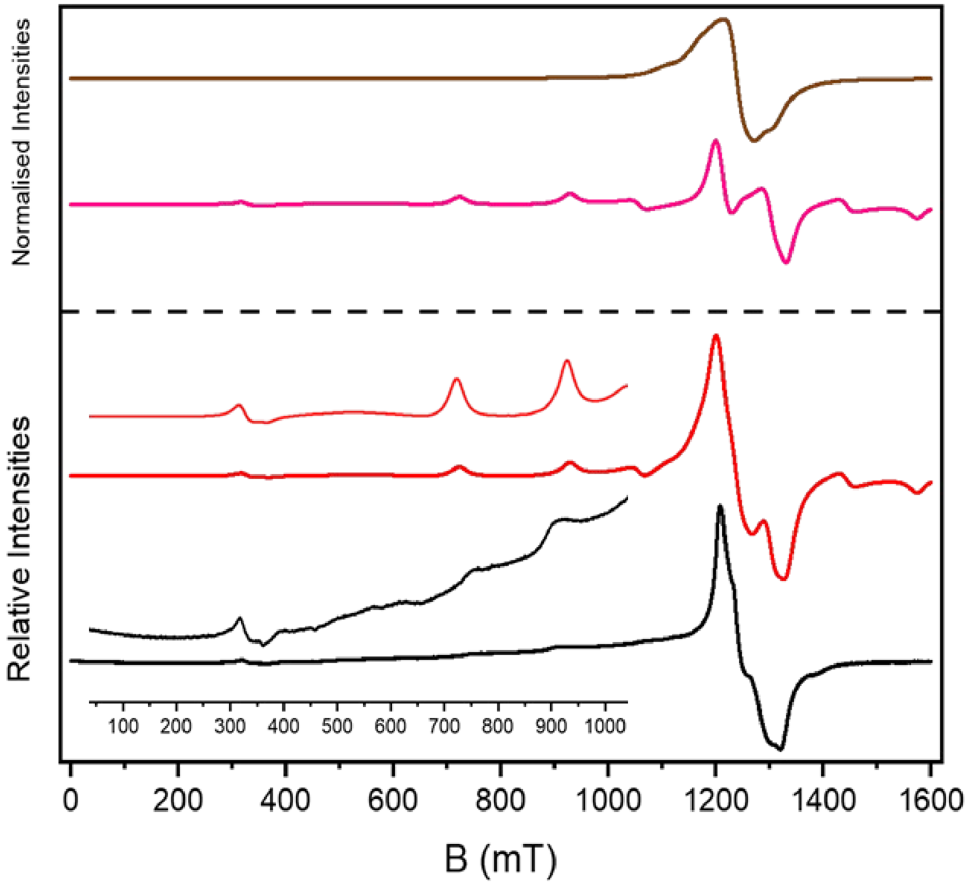

Figure S1: CW Q-band (ca. 34 GHz) EPR spectrum of **1** as a powder at 5 K (black) and simulation (blue). The break-down of the simulations and parameters used are as in Figure 3. Insert shows ow field section at higher receiver gain. The experiment was performed with a frequency of 34.092926 GHz.

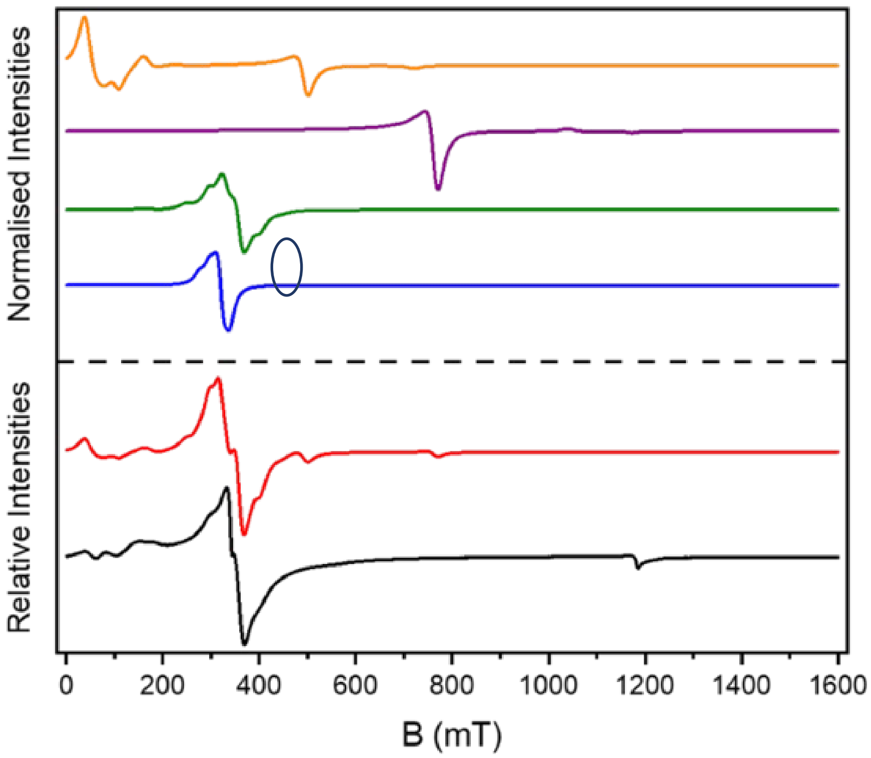

Figure S2: CW X-band (ca. 9.5 GHz) EPR spectrum of **2** as a powder at 5 K (black) and simulation (red). The break-down of the simulation for each spin state (above the dashed line) is system 3 (*S* = ½, isolated Cu(II), blue), system 4 (*S* = 2, green), system 5 (*S* = 1, purple) and system 6 (*S* = 1, orange). The signal from O_2 is in the dashed blue oval. The experiment was performed with a frequency of measured at 9.388738 GHz.

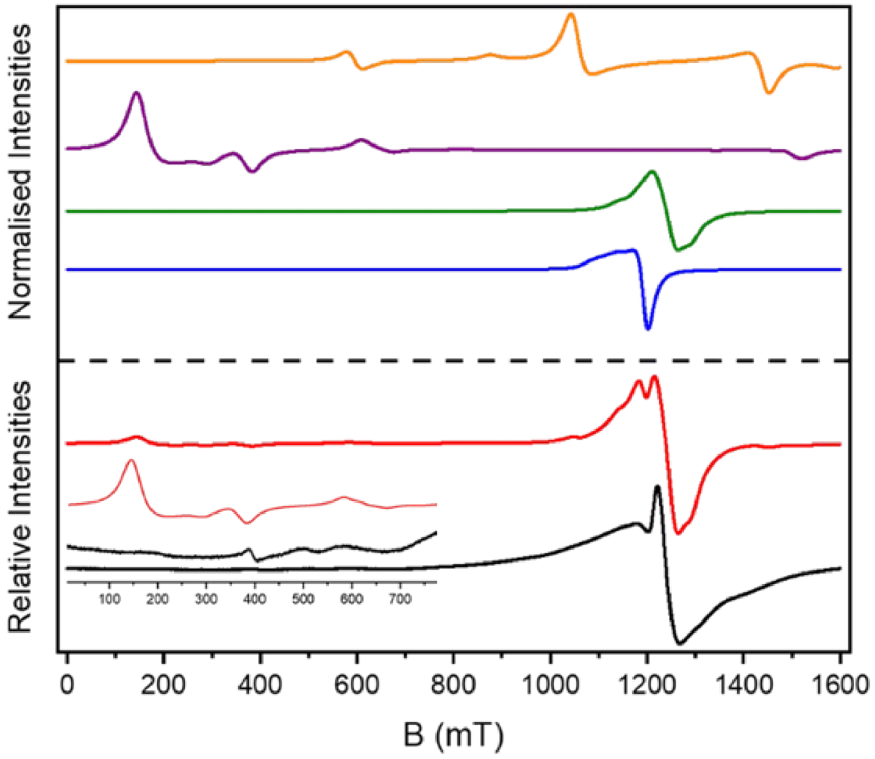

Figure S3: CW Q-band (ca. 34 GHz) EPR spectrum of **2** as a powder at 5 K (black) and simulation (red). The break-down of the simulations and parameters used are as in Figure 4. The experiment was performed with a frequency of 34.120297 GHz.

SI References:

S1. Rigaku Oxford Diffraction, CrysAlisPro Software system, version 40, Rigaku Corporation, Oxford, UK, **2017**.

S2. G. M. Sheldrick, *Acta Cryst.* A, **2015**, *71*, 3-8.

S3. O.V. Dolomanov, L. J. Bourhis, R. J. Gildea, J. A. L. Howard, H. Puschmann *J. Appl. Cryst.* **2009**, *42*, 339-341.

S4. J. Jaklic, P. Prelovšek, *Phys. Rev. B* **1994**, *49*, 5065.

S5. J. Schnack, O. Wendland, *Eur. Phys. J. B* **2010**, *78*, 535.

S6. S. Stoll, A. Schweiger, *J. Magn. Reson*. 2006, **178**, 42-55.
